# Tick-Borne Transmission of Murine Gammaherpesvirus 68

**DOI:** 10.3389/fcimb.2017.00458

**Published:** 2017-10-31

**Authors:** Valeria Hajnická, Marcela Kúdelová, Iveta Štibrániová, Mirko Slovák, Pavlína Bartíková, Zuzana Halásová, Peter Pančík, Petra Belvončíková, Michaela Vrbová, Viera Holíková, Rosemary S. Hails, Patricia A. Nuttall

**Affiliations:** ^1^Biomedical Research Center, Institute of Virology, Slovak Academy of Sciences, Bratislava, Slovakia; ^2^Institute of Zoology, Slovak Academy of Sciences, Bratislava, Slovakia; ^3^Department of Microbiology and Virology, Comenius University, Bratislava, Slovakia; ^4^Centre for Ecology and Hydrology, Wallingford, United Kingdom; ^5^Department of Zoology, University of Oxford, Oxford, United Kingdom

**Keywords:** gammaherpesvirus, MHV68, transmission, tick, *Ixodes ricinus*, arbovirus

## Abstract

Herpesviruses are a large group of DNA viruses infecting mainly vertebrates. Murine gammaherpesvirus 68 (MHV68) is often used as a model in studies of the pathogenesis of clinically important human gammaherpesviruses such as Epstein-Barr virus and Kaposi's sarcoma-associated herpesvirus. This rodent virus appears to be geographically widespread; however, its natural transmission cycle is unknown. Following detection of MHV68 in field-collected ticks, including isolation of the virus from tick salivary glands and ovaries, we investigated whether MHV68 is a tick-borne virus. Uninfected *Ixodes ricinus* ticks were shown to acquire the virus by feeding on experimentally infected laboratory mice. The virus survived tick molting, and the molted ticks transmitted the virus to uninfected laboratory mice on which they subsequently fed. MHV68 was isolated from the tick salivary glands, consistent with transmission via tick saliva. The virus survived in ticks without loss of infectivity for at least 120 days, and subsequently was transmitted vertically from one tick generation to the next, surviving more than 500 days. Furthermore, the F1 generation (derived from F0 infected females) transmitted MHV68 to uninfected mice on which they fed, with MHV68 M3 gene transcripts detected in blood, lung, and spleen tissue of mice on which F1 nymphs and F1 adults engorged. These experimental data fulfill the transmission criteria that define an arthropod-borne virus (arbovirus), the largest biological group of viruses. Currently, African swine fever virus (ASFV) is the only DNA virus recognized as an arbovirus. Like ASFV, MHV68 showed evidence of pathogenesis in ticks. Previous studies have reported MHV68 in free-living ticks and in mammals commonly infested with *I. ricinus*, and neutralizing antibodies to MHV68 have been detected in large mammals (e.g., deer) including humans. Further studies are needed to determine if these reports are the result of tick-borne transmission of MHV68 in nature, and whether humans are at risk of infection.

## Introduction

Although all vertebrates are probably infected with at least one herpesvirus species, herpesviruses are believed not to infect arthropods, and vector-mediated transmission of herpesviruses is unreported hitherto (King et al., 2011). Generally, the natural host range of individual herpesviruses is very restricted and they are highly adapted to their hosts. Most herpesviruses establish a systemic infection following a cell-associated viremia during primary infection. Severe infection is usually observed only in the very young, the fetus, the immunocompromised, or following infection of an alternative host. The key to survival of herpesviruses is their ability to establish life-long latent infections (Roizman and Pellett, 2001; Adler et al., 2017). Members of the subfamily *Gammaherpesvirinae*, which includes murid herpesvirus 4 (MuHV-4, more commonly known as murine gammaherpesvirus 68, MHV68) in the genus *Rhadinovirus*, establish latent infections in lymphocytes or lymphoid tissue. Persistent human gammaherpesvirus infection is associated with the development of malignancies such as Burkitt's lymphoma, Hodgkin's disease, and Kaposi's sarcoma (Mesri et al., 2014). Epstein-Barr virus (EBV) and Kaposi's sarcoma-associated herpesvirus (KSHV) are host (human)-specific and therefore lack a tractable *in vivo* infection model (Cieniewicz et al., 2016; Habison et al., 2017). The discovery of MHV68 provided what is now a much studied laboratory model for investigating virus reactivation from latency as well as host mechanisms of immune control, and the genetic basis of viral fitness in different cell types and tissues (Rajčáni and Kúdelová, 2007; Sattler et al., 2016).

MHV68 was originally isolated from the bank vole *Myodes glareolus* (formerly *Clethrionomys glareolus*) during a study on the ecology of arboviruses in Slovakia (Blaskovic et al., 1980). Four other herpesviruses were isolated at the same time, two from bank voles and two from the yellow-necked field mouse *Apodemus flavicollis*. At least eight isolates of MHV68 have been recorded (Mistríková et al., 2000a). It has been speculated this group of viruses is geographically widespread and may occur throughout the mouse and vole subfamilies although a distinct wood mouse herpesvirus (classified as MuHV-7) has been isolated from *Apodemus sylvaticus* and bank voles, while a different gammaherpesvirus infects house mice, *Mus musculus* (Nash et al., 2001; Ehlers et al., 2007; Hughes et al., 2010a).

Little is known of the natural history of MHV68 (Nash et al., 2001; Telfer et al., 2007; Knowles et al., 2012). Antibodies against MHV68 have been detected in sera of at least 13 different mammalian species including large mammals (e.g., deer, sheep) that share the biotope of infected rodents (Mistríková et al., 2000a,b). Neutralizing antibodies to MHV68 have also been detected in humans but they are considered to reflect antigenic cross-reactions with human gammaherpesviruses (Hricová and Mistríková, 2007). Experimental infection with MHV68 is typically via the respiratory route although no evidence was found for air-borne or contact transmission between either female bank voles or female laboratory mice in experimental studies (François et al., 2010). However, transmission has been demonstrated experimentally in laboratory mice through breast milk to offspring and sexually from females to males (Raslova et al., 2001; François et al., 2013; Zeippen et al., 2017). The significance of these findings for the transmission cycle needs to be determined especially as the pathogenesis of MHV68 in natural rodent hosts differs from laboratory mice (François et al., 2010; Hughes et al., 2010b).

Recently published reports of infectious MHV68 in the salivary glands, intestines and ovaries of wild caught questing *Dermacentor reticulatus* adult ticks, and MHV68 DNA detection in nymphs of *Ixodes ricinus* ticks infesting green lizards (*Lacerta viridis*) and in questing adult *Haemaphysalis concinna*, raise the question of whether ticks can act as transmission vectors of MHV68 (Ficová et al., 2011; Kúdelová et al., 2015; Vrbová et al., 2016). In Europe, rodents are important hosts for immature stages of *I. ricinus* and adult *D. reticulatus*, vectors of human pathogens such as tick-borne encephalitis virus (TBEV) and the Lyme disease spirochete, *Borrelia burgdorferi*. These tick-borne pathogens circulate among small rodents and larger mammals through horizontal transmission from infected tick to uninfected vertebrate host and *vice versa*. They replicate in both vertebrates and ticks, demonstrating biological transmission, which distinguishes them from mechanically transmitted pathogens that are carried, for example, on the mouthparts of insect vectors (Kuno and Chang, 2005).

To test whether ticks can act as vectors of MHV68, we determined the ability of *I. ricinus* to acquire and subsequently transmit MHV68 using the laboratory mouse as the vertebrate host. First, we established whether MHV68 is transmitted by *I. ricinus* following simple injection of ticks with virus and then allowing the ticks to feed on uninfected mice. Second, we tested whether MHV68 can be transmitted from an infected mouse to uninfected ticks, survive trans-stadially, and subsequently be transmitted by the molted ticks. Third, we examined whether MHV68 can be transmitted vertically from one tick generation to the next by injecting female ticks with MHV68, allowing them to feed and lay eggs, feeding the emergent F1 larvae on uninfected mice, and then testing the ability of the F1 nymphs and F1 adults to transmit the virus when feeding on uninfected mice. The results provided evidence of MHV68 trans-stadial survival in ticks (with some pathogenesis), effective tick-borne virus transmission to uninfected mice, efficient vertical transmission of MHV68 to F1 ticks, and the remarkable ability of MHV68 to survive >500 days from infected adult females through to the subsequent F1 adult tick population. The experimental results together with published data on detection of MHV68 in field caught ticks and serological evidence of infection of a variety of mammals, strongly suggest that MHV68 is circulating in nature between mammals and ticks, although they do not preclude additional direct routes of transmission between rodents.

## Materials and methods

### Ethics statement

All animals used in this study were housed and handled according to the statute of the Slovak Republic (No. 23/2009) for care of experimental animals. The protocol for using ticks and mice was approved by the State Veterinary and Food Administration of the Slovak Republic (Permit Number: 1018/09-221, 292/16-221c).

### Virus

MHV68 originally isolated from *M. glareolus* (Blaskovic et al., 1980) was kindly provided by Prof. Mistríková (Comenius University, Bratislava, Slovakia). MHV68 was subsequently twice plaque-purified to obtain clone f2.6 as described previously (Macáková et al., 2003). Virus was propagated and titrated using Baby hamster kidney (BHK-21) fibroblasts (ATCC number: CCL-10) as described previously (Raslova et al., 2001). Cell cultures were maintained in Dulbecco's Minimum Essential Medium (Gibco) supplemented with 10% (v/v) fetal bovine serum (FBS), 2 mM glutamine (Invitrogen) and penicillin–streptomycin–amphothericin (Cambrex) at 37°C.

### Tick maintenance

*I. ricinus* originated from ticks collected by dragging the vegetation in areas around Bratislava known to be free of TBEV. The ticks have been maintained as a colony at the Institute of Zoology for several generations by feeding on rabbits and mice. Non-feeding ticks are kept in a desiccator containing a saturated solution of MgSO_4_, at 23 ± 3°C, 90–95% RH, on a light:dark cycle of 16:8 h.

### Injection of ticks with MHV68

Hollow glass needles with a microscopic tip were prepared from borosilicate glass capillary tubes (O.D.: 1.5 mm, I.D.: 0.86 mm, 10 cm length) by means of a P-30 Micropipette puller (Sutter Instrument Company, USA). Unfed female ticks were then microinjected with 1.25 × 10^4^ PFU of MHV68 (0.5 μl) under a stereo zoom microscope (Wild M 400, Wild Heerbrugg AG, Switzerland) through the coxal plate of the second pair of legs using a digital microinjector ™ system (MINJ-D-CE; Tritech Research, Inc.; USA). Clean nitrogen served as a gas source to produce an injection pressure of 20 psi (= app. 1.38 bar). The injection interval was set to 1.0 s. Following injection, the ticks were placed into a desiccator and incubated at room temperature.

### Infection of animals

For virus transmission studies, outbred ICR (Institute of Cancer Research) female mice, 6–8 weeks of age, were used. Mice were lightly anesthetized with halothane and exposed intranasally to 2 × 10^5^ PFU MHV68 in 25 μl of sterile phosphate-buffered saline (PBS). The virus was inoculated onto the external nares with a micropipette and the animal observed to ensure the drop containing the virus was inhaled. The animals were then transferred to a different room where they were housed individually using the mouse Blue Line IVC Sealsafe caging system (TECNIPLAST, Italy) to avoid the possibility of air-borne transmission of infection.

### Tick infestation of mice

Five days post exposure of mice to MHV68, each animal was infested with uninfected larvae (70–100 larvae/mouse) or nymphs (20 nymphs/mouse). Immature stages of ticks were retained on animals within a short piece of neoprene tube with the open end covered in nylon gauze to prevent the ticks from escaping. Groups of adult ticks, two females and two males (to promote female tick feeding), were placed in plastic chambers. Feeding chambers were attached to the back of mice using a non-irritating, adhesive latex glue, Pritt-Copydex (Henkel). Ticks were allowed to feed to repletion. Naturally detached engorged ticks were recovered twice daily from the feeding chambers of the infested animals and removed to a desiccator at room temperature and 90–95% relative humidity to allow molting. Engorged ticks were maintained for development into nymphs or adults for use in the study.

### Immunofluorescence assay to detect infectious virus cultured from ticks

Four adult female ticks, 120 days after injection with MHV68, and two control uninfected ticks were cleansed several times in PBS containing antibiotics, and then individually homogenized using a sterile mortar under sterile conditions not used for virus manipulation. The homogenate of each individual tick was clarified by centrifugation (2 min at 10,000 × g) and suspended in cultivation medium. Supernatant of each tick was used for determination of the presence of live virus by replication in a mammalian cell line. BHK-21 cells were grown in L-15 medium, supplemented with 10% FBS, and 1% antibiotic/antimycotic solution (Sigma). Cells were inoculated onto glass coverslips, 1.6 × 10^5^ cells per well in six-well plates. After 24 h incubation, the media were exchanged and cells were incubated for 90 min in fresh cultivation medium containing the homogenized tick supernatant. Each coverslip culture was inoculated with supernatant derived from a single tick. Media were exchanged again and cells were cultivated in fresh media for 96 h. After washing twice in PBS, the cells were fixed in acetone (−20°C) for 5 min and incubated in blocking buffer (1% BSA in PBS) for 30 min. Cells were then incubated in rabbit anti-MHV68 polyclonal antibody (dilution 1:200 in PBS + 1% BSA, 60 min), kindly provided by Dr. Rajčáni, Institute of Virology SAS (Rajcáni et al., 1985). After three washes with PBS + 0.2% Tween-20, cells were stained with Alexa Fluor 488 goat anti-rabbit IgG antibody (Invitrogen, USA) (dilution 1:2,000 in PBS + 1% BSA) for 60 min. Finally, the cells were washed three times in PBS. Coverslips were mounted in mounting medium and cells were examined under a fluorescence microscope.

### Immunofluorescence assay to detect virus in tick tissues

Two female *I. ricinus* ticks (F1 generation) partially fed on uninfected mice for 4 days were examined for viral antigen. Ticks were opened at the posterior part of their opisthosoma using a razor blade, embedded in tissue freezing medium OCT (Leica Biosystem, Germany), snap-frozen and stored at −80°C until examination. Semi-thin sections (6 mm) were obtained using a cryostat (Leica, Germany), collected on Superfrost plus microscope slides (Menzel-Gläser, Thermo Scientific), air dried and stored at −80°C. Sections were fixed and permeabilized with ice cold acetone for 10 min, air dried and rinsed three times in PBS for 5 min each. To reduce background, slides were incubated in blocking buffer (PBS/1% BSA/0.05% Tween20) for 1 h at RT. After washing in PBS/Tween 20, sections were incubated with rabbit anti-MHV68 polyclonal antibody (dilution 1:200 in PBS + 1% BSA, 60 min) kindly provided by Dr Rajčáni, Institute of Virology SAS (Rajcáni et al., 1985) for 1 h at 37°C. To test for nonspecific binding to immunoglobulin binding proteins in tick salivary glands, we used rabbit polyclonal serum against PB1-F2 protein of influenza virus A (H1N1) (kindly provided by Department of Orthomyxoviruses of Institute of Virology SAS) as a negative control. Goat anti-rabbit IgG conjugated with Alexa 488 (Invitrogen) was used as a secondary antibody. The sections were examined under fluorescence microscope (Zeiss Axiovert 40 CFL).

### Isolation of total DNA from ticks and murine samples

Salivary glands of ticks examined for the presence of MHV68 DNA were dissected and their total DNA was isolated using QIAamp DNA Mini Kit (Qiagen, USA) as described previously (Kúdelová et al., 2015). DNA content in samples was measured using a spectrophotometer and then immediately assayed by nested PCR. Mice were tested for the presence of MHV68 DNA in their blood, lung, and spleen at 15 days post-infestation. Blood samples of 0.2–0.5 ml were collected from the orbital sinus of each halothane-anesthetized mouse. DNA from the blood was isolated using QIAamp DNA Blood Mini Kit (Qiagen, USA). DNA from murine lung (25 mg) and spleen (25 mg) tissue collected at 15 days post-infestation was isolated using QIAamp DNA Mini Kit (Qiagen, USA) and measured as above.

### Isolation of total RNA from murine blood, lung, and spleen samples

Samples of 100 μl blood, 25 mg lung tissue, and 25 mg spleen tissue were processed. Total RNA from murine blood was isolated using Spin Column total RNA mini preps Super kit (Bio Basic) and from murine spleen and lungs using TRI-Reagent (Sigma) following the manufacturer's instructions. Prior to testing, 1.5–2 μg total RNA sample was treated with 1 U DNase I (RNase-free) (Novagen) in the presence of 50 mM Tris-HCl (pH 8.3), 75 mM KCl, 15 mM MgCl_2_, and 10 mM DTT reaction buffer for 60 min and the treatment was terminated by the addition of 1 μl 25 mM EDTA and heat-inactivation at 65°C for 15 min. An aliquot of DNase I-treated RNA of each sample was tested by nested PCR to confirm there was no viral DNA contamination in the RNA samples.

### Detection of MHV68 by nested PCR assay

MHV68 presence was examined in tick salivary glands and blood, lungs, and spleen of mice by nested PCR developed by Kúdelová et al. (2015) with some modifications. Briefly, DNA purified from tick salivary glands (1.5 μg), or from murine blood, lungs, or spleen (100 ng), was used to amplify MHV68 genomic *ORF* (open reading frame) *50* gene of MHV68 (in the virus genome from 61,907 to 69,373 nt) encoding the Replication and Transcription Activator (Rta) (Acc. No. AF105037).

Outer primers of the first PCR (*ORF50*F1: 5′-AACTGGAACTCTTCTGTGGC-3′ and *ORF50*R1: 5′-GGCCGCAGACATTTAATGAC-3′) generated a 586-bp product (Kúdelová et al., 2015). The reaction mixture contained 50 mM KCl, 10 mM Tris-HCl (pH 8.5), 0.1% Triton X-100, 1.5 mM MgCl_2_, 0.3 mM nucleotides, 0.3 mM of each primer, and 1 U of Go*Taq* polymerase (Promega) in a 25-μl total volume. PCR program was as follows: 45 cycles of 94°C for 30 s, 60°C for 30 s, and 72°C for 30 s, followed by extension at 72°C for 5 min. The second round of PCR was performed using 1–3 μl of the first-round PCR as a template in the same reaction mixture except that inner PCR primers were used (*ORF50*F2: 5′-CCCCAATGGTTCATAAGTGG-3′ and *ORF50*R2: 5′-ATCAGCACGCCATCAACATC-3′), which generated a 382-bp product. The conditions for the nested PCR were identical except that 30 cycles were used.

Second-round PCR products were resolved by SYBR Green staining (BioRad) on 1% agarose gels. Molecular weights of PCR products were estimated by comparison with HyperLadderI (Bioline) and/or GeneRuler™ 100 bp Plus DNA ladder (Fermentas). The MHV68 BAC DNA, kindly provided by Prof. Ulrich H. Koszinowski (Adler et al., 2000), was used to check the sensitivity of the nested PCR for detection of MHV68 DNA in the presence of different amounts of template. MHV68 BAC DNA was quantified spectrophotometrically and diluted in mouse liver DNA (5 mg/ml) in TE buffer. Either 1.5 μg or 100 ng total nucleic acid from serial 10-fold dilutions of MHV68 BAC DNA (from 10^6^ to 0.1 copies) in mouse liver DNA were analyzed by nested PCR; MHV68 BAC DNA served as a positive control. DNA purified from salivary glands of uninfected ticks, and the blood, lungs, and spleen of uninfected mice, and purified water, served as negative controls. Assays with 1.5 μg of DNA demonstrated approximately single-copy sensitivity reported previously for tick samples (Kúdelová et al., 2015), with no false-positive results. PCR assays with 100 ng of template demonstrated sensitivity levels of about 40 copies of genome, with no false-positive results. Samples that tested positive were re-examined using a nested PCR specific for the glycoprotein gp150 gene of MHV68 originally developed to detect virus in free-living rodents (Klempa et al., 2001) and previously described in detail (Ficová et al., 2011).

### Sequencing of PCR products

Nested PCR products amplified from DNA isolated from the blood of three MHV68 positive mice and salivary glands of two MHV68 positive ticks were purified with Wizard® SV Gel and PCR Clean-up System (Promega, USA) according to the manufacturer's instructions. They were sequenced using both inner primers for the *ORF50* gene specific PCR reaction (see above) using a commercial sequencing service (BITCET). The sequences were aligned and compared with the *ORF50* sequence of MHV68 (Acc. No. AF105037).

### Viral genome quantification

Viral genome loads were measured by quantitative real time PCR to detect *ORF65* which encodes the capsid protein, M9 (Guo et al., 2009). DNA from murine blood, lungs, and spleen tissue (100 ng) was used to amplify MHV68 genomic coordinates 94,119 to 94,184 within *ORF65* gene using StepOne Real-Time PCR System (Applied Biosystems). Primers *ORF65*F: 5′-GTC AGG GCC CAG TCC GTA-3′ and *ORF65*R: 5′-TGG CCC TCT ACC TTC TGT TGA-3′) overlapped a 65 bps long fragment. PCR mixture contained 0.5 μM of each primer and Maxima SYBR Green PCR reaction buffer with ROX-passive reference dye (Thermo Scientific) in the final volume of 20 μl. PCR program was as follows: 40 cycles of 94°C for 15 s, 58°C for 15 s, and 72°C for 15 s. qPCR standard curve was established using 10-fold serial dilutions of the MHV68 BAC DNA; 10^6^ to 10^0^ copies were used as templates in PCR mixtures that were amplified in parallel. Specificity of qPCR products was confirmed by melting curve analyses. PCR products were quantified by comparison with the standard curve. Viral genome equivalents in 1 ml of blood or 1 mg of tissue were determined from the mean of triplicate real-time PCR assays for each sample.

### Detection of MHV68 *M3* gene transcripts

Transcription of the early-late *M3* gene of MHV68 (GenBank accession number U97553, coordinates 6,060–7,277) was detected by nested RT-PCR. Prior to use, total RNA purified from murine blood, lungs and spleen tissue (100 ng) was treated with DNAse I according to the manufacturer's instructions. RNA purified from organs of uninfected mice infested with uninfected ticks, served as negative controls. One microgram RNA was used for the reverse transcription in the reaction mixture with 250 ng of random primers (Promega) and 10 mM each dNTP incubated at 65°C for 5 min folowed by cooling in ice. Then, the first-strand buffer (Invitrogen) with 10 mM DTT were added to final volume of 19 μl. Prior to adding 200 U of M-MLV Reverse Transcriptase (Invitrogen), the reaction mixture was incubated at 37°C for 2 min, followed by incubation at 25°C for 10 min, then 37°C for 50 min, and final heat-inactivation of enzyme at 70°C for 15 min. Fifty nanograms of cDNA was used to amplify a 520 bp region of the *M3* gene (genome coordinates 6075-6594). Reaction mixture in a total volume of 25 μl contained 0.5 μM of each outer primer (*M3P*F1: 5′-ACT CCA GCC TGT ACT GTT GC-3′and *M3P*R1: 5′-TCT GCC CCA CAA CCA AGT TT-3′), 50 mM KCl, 10 mM Tris-HCl (pH 8.5), 0.1% Triton X-100, 1.5 mM MgCl_2_, 0.2 mM dNTPs, and 1 U Go*Taq* polymerase (Promega). PCR program of the first PCR was as follows: 35 cycles of 94°C for 40 s, 59.3°C for 30 s, and 72°C for 45 s. The second round of PCR was performed using 1 μl of the first-round PCR as a template in the same reaction mixture except that inner PCR primers were used (*M3P*F2: 5′-ACT GGC CCT CAA CCA GTC TA-3′ and *M3P*R2: 5′-TAC AAG TAC AGC GTG AGC CC-3′) that generated a 241 bp product (genome coordinates 6171-6411). Conditions for the nested PCR were identical except that annealing temperature (59.8°C) and cycling conditions (40 cycles).

## Results

### Tick-borne virus transmission following artificial infection of ticks by injection

**Figure 1:**
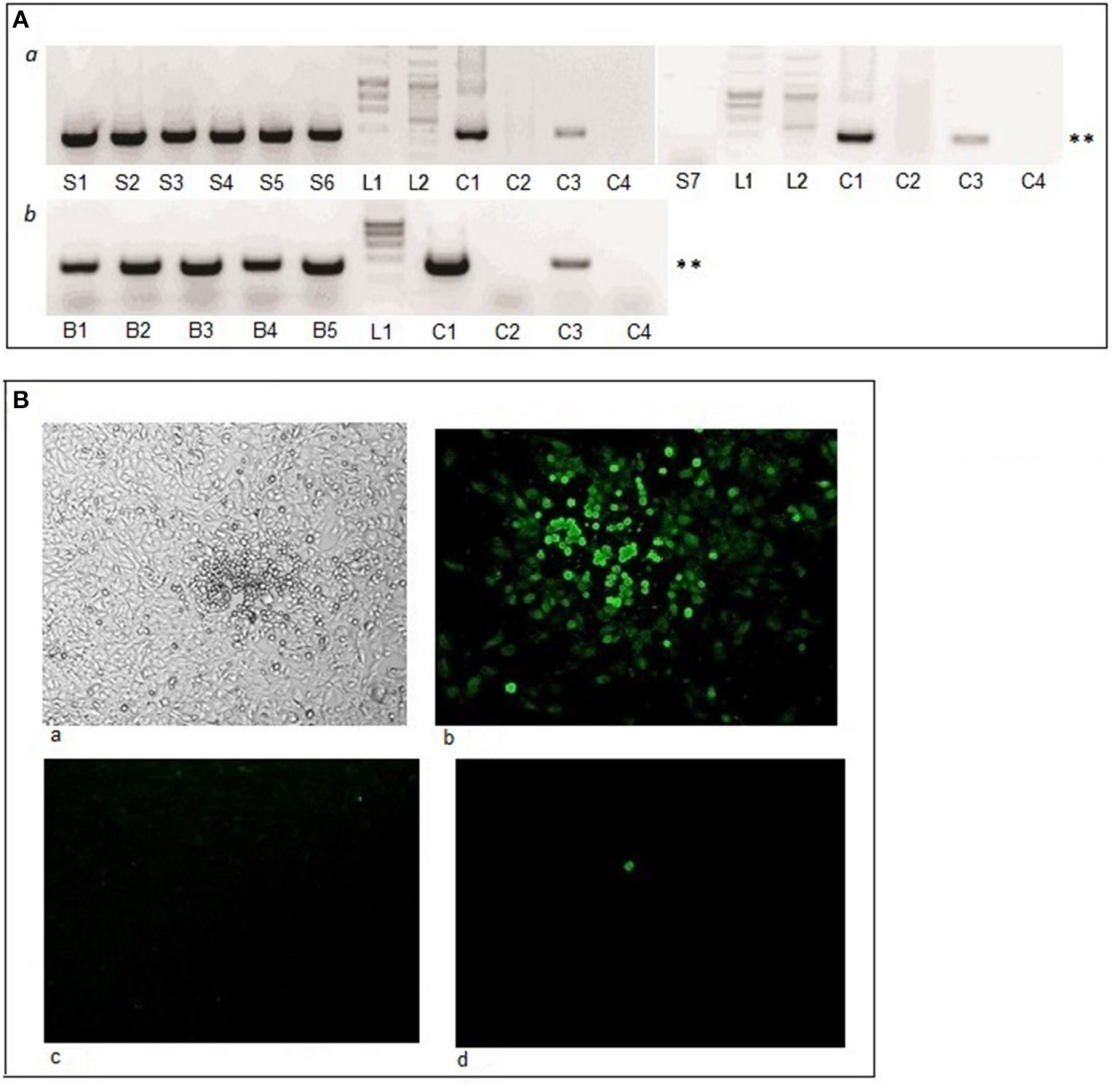
Tick-borne virus transmission and tick infection following injection of ticks with MHV68. **(A)** Virus detected by nested PCR. Lanes S1-S7, salivary glands of virus-injected ticks fed on uninfected mice for 5 days; lanes B1-B5, blood samples of five mice 15 days after tick infestation; L1, HyperLadder; L2, GeneRuler DNA ladder; lane C1, MHV68 DNA (positive control); lane C2, nested PCR without template (negative control); lane C3, MHV68 DNA nested PCR 1st round product with outer primers (positive control); lane C4, 1st PCR round with outer primers without template (negative control). ^**^ Indicates MHV68 *ORF50* gene PCR product of 382 base pairs. **(B)** Infectivity as determined by plaque formation in BHK-21 cells. Cells inoculated with homogenate of virus infected tick and observed by **(a)** light microscopy (magnification x50) and **(b–d)** specific immunofluorescence staining (magnification x200). **(b)** Single plaque shown of a maximum 5 plaques observed per tick; **(c)** control, uninfected cells; and **(d)** cells inoculated with homogenate of uninfected tick.

To test whether MHV68 can survive in ticks and be transmitted during blood-feeding, unfed adult female ticks were inoculated with 0.5 μl PBS containing 1.25 × 10^4^ PFU (plaque-forming units) of MHV68 using a digital microinjector system. The inoculation was through the coxal plate of the second pair of legs as this route minimizes the chance of damaging internal organs, and allows the virus to enter directly into the hemolymph of the tick and be disseminated throughout the hemocoel. Following injection, the ticks were placed in a desiccator and maintained at room temperature and 90–95% relative humidity. Two weeks after they had been inoculated with MHV68, the female ticks were allowed to infest uninfected mice: 2 inoculated females together with 2 uninfected male ticks (to improve female blood-feeding) per mouse. Five days later, when the ticks were partially engorged, a total of six female ticks were removed from three mice and their salivary glands dissected out and assayed using a nested PCR targeting MHV68 *ORF50*. All salivary glands prepared from these six injected ticks were MHV68 positive (Figure 1A, *a*). Sequencing results of the PCR products shown in lanes 1 and 3 revealed respectively 99.8 and 100% identity with the *ORF50* gene of MHV68.

Having shown that MHV68 DNA can survive in ticks and access the salivary glands following artificial inoculation into the tick hemocoel, we then examined whether the virus is transmitted by the injected ticks to uninfected mice (Table 1, Experiment 1). Female ticks injected with MHV68 were allowed to feed to repletion on 5 uninfected mice, 2 inoculated females together with 2 uninfected male ticks per mouse. Blood samples were collected from the mice on day 15 post-infestation and tested by nested PCR; all five mice were MHV68 positive (Figure 1A, *b*). Sequencing of the PCR product shown in lane 5 revealed 100% identity with MHV68 *ORF50*, indicating that the virus detected in the blood was the same as the virus injected into the ticks that fed on the mice.

**Table 1:** Artificial (injection) and natural (blood-feeding) acquisition and blood-feeding transmission of MHV68 by ticks.

**Experiment**	**Virus acquisition**		**Virus transmission**	
	**Ticks**	**Tick treatment**	**Molted ticks**	**Number (%) mice virus positive^a^**
1	Adults	Injected with MHV68 and fed on uninfected mice	n. a.	5/5 (100%)
1 (control)	Adults	Untreated and fed on uninfected mice	n.a.	0/2
2	Larvae	Fed on MHV68 infected mice	Nymphs	2/7 (28.6%)
2 (control)	Larvae	Fed on uninfected mice	Nymphs	0/2
3	Nymphs	Fed on MHV68 infected mice	Adults	3/11 (27.3%)
3 (control)	Nymphs	Fed on uninfected mice	Adults	0/1
4	Nymphs	Fed on MHV68 infected mice	Adults	2/13 (15.4%)
4 (control)	Nymphs	Fed on uninfected mice	Adults	0/2

*n.a., not applicable*.

a *Positive by nested PCR of blood collected 15 days post-infestation*.

To examine the persistence of infectious MHV68 in ticks, whole body extracts were prepared from 4 of the ticks, 120 days after virus injection, together with 2 control ticks, and inoculated onto monolayers of BHK-21 cells. A few plaques (2–5) became visible after 3 days of culture, which were positive by immunofluorescence staining using specific anti-MHV68 polyclonal antibody (Figure 1B). Infectious virus was detected in extracts of all four ticks; all controls were negative. Because the ticks were unfed, the small number of plaques does not reflect the amount of virus transmitted as tick-borne virus replication occurs during tick feeding (Kaufman and Nuttall, 2003).

### Tick-borne virus transmission following natural infection of ticks by blood-feeding

**Figure 2:**
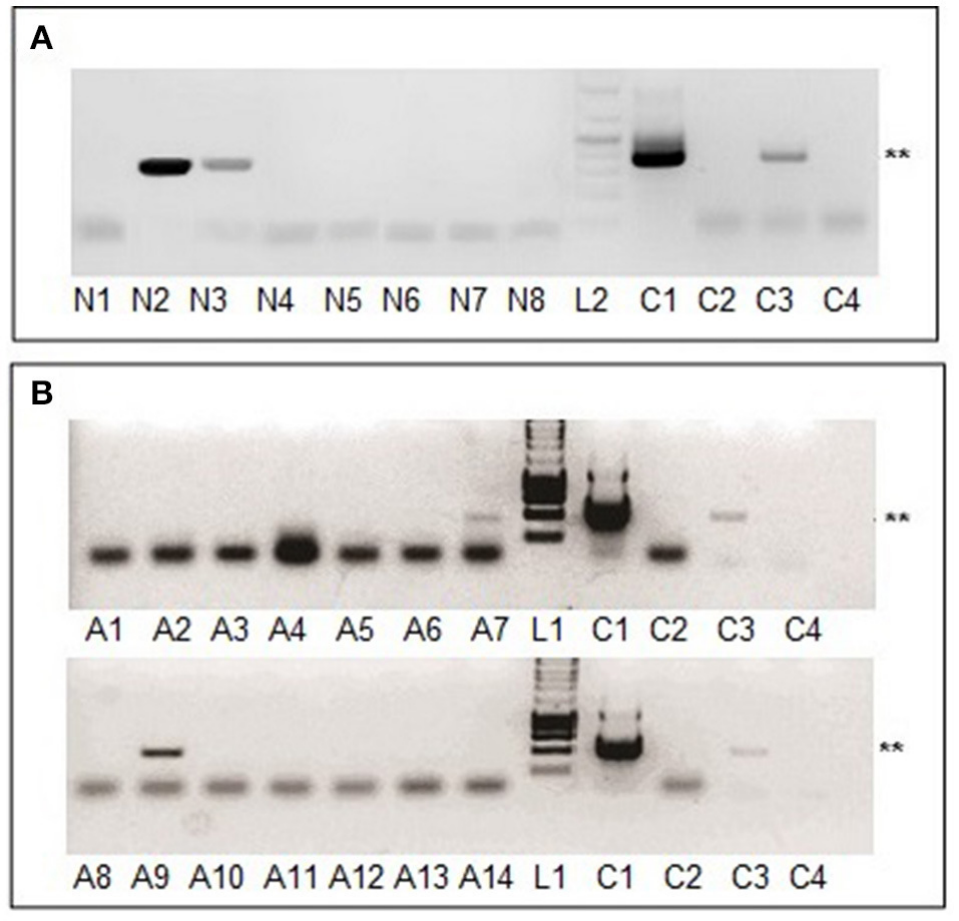
MHV68 detection in blood samples from mice infested with naturally infected nymphs or adult ticks. **(A)** Lanes N1-N7, blood samples of mouse 1-7 exposed to nymphs molted from larvae that had engorged on infected mice; lane N8, blood of mouse infested with uninfected nymphs. **(B)** Lane A1, blood of mouse infested with uninfected adult ticks; lanes A2-A14, blood samples of 13 mice exposed to adults molted from nymphs that had engorged on infected mice. All blood samples were collected 15 days after tick infestation. Lanes L1, L2, C1–C4 as for Figure 1A. ^**^Indicates MHV68 ORF50 gene PCR product of 382 base pairs.

Having demonstrated MHV68 transmission to mice by virus-injected ticks, we determined whether ticks infected via the natural route (by feeding on infected mice) can transmit MHV68. Unlike blood-feeding insects, engorged immature ticks molt before feeding again; hence, transmission ability was tested after tick molting. In Experiment 2 (Table 1), larvae were allowed to feed on mice that had been infected 5 days previously by intranasal exposure to MHV68. Following engorgement (completed in 2–4 days) and drop off, the larvae were maintained under conditions that allowed molting to nymphs. The emergent nymphs, 117 days after completing engorgement on infected mice as larvae, were then fed on seven uninfected mice. Two uninfected mice infested with uninfected nymphs served as negative controls. On day 15 post-infestation, when the last ticks to reach engorgement had dropped off, blood samples from the mice were collected and assayed for the presence of viral DNA. Two of seven mice (28.6%) were found to be MHV68 positive (Figure 2A). The results were confirmed using different template inputs in the PCR (specific for the gp150 gene; see Materials and Methods). Sequencing of the PCR products from one positive mouse (Figure 2A, lane 2) revealed 99.8% sequence identity with MHV68 *ORF50*. The results were confirmed using the same template but with a different primer set in a separate PCR specific for the gp150 gene (see Materials and Methods).

Similarly designed experiments were undertaken using uninfected nymphs as the starting point (Table 1, Experiment 3). Nymphs were fed (4–6 days) on experimentally infected mice, collected after drop off, and then allowed to molt to adults. Emergent adults, 130 days after completing engorgement on infected mice as nymphs, were allowed to feed on 11 uninfected mice (2 males and 2 females per mouse). One uninfected mouse infested with uninfected adults served as a negative control. Blood samples were obtained from infested animals 10–12 days after engorged ticks dropped off the mice (15 days post-infestation). Three of 11 mice (27.3%) were MHV68 positive (data not shown). In a repeat experiment (Table 1, Experiment 4), adults metamorphosed from nymphs were fed on 13 mice of which two (15.4%) were MHV68 positive, 15 days post-infestation (Figure 2B, A7 and B2). Sequencing results of sample B2 revealed 99.8% identity of the PCR product compared with MHV68 *ORF50*.

### Vertical transmission of MHV68 in ticks

**Figure 3:**
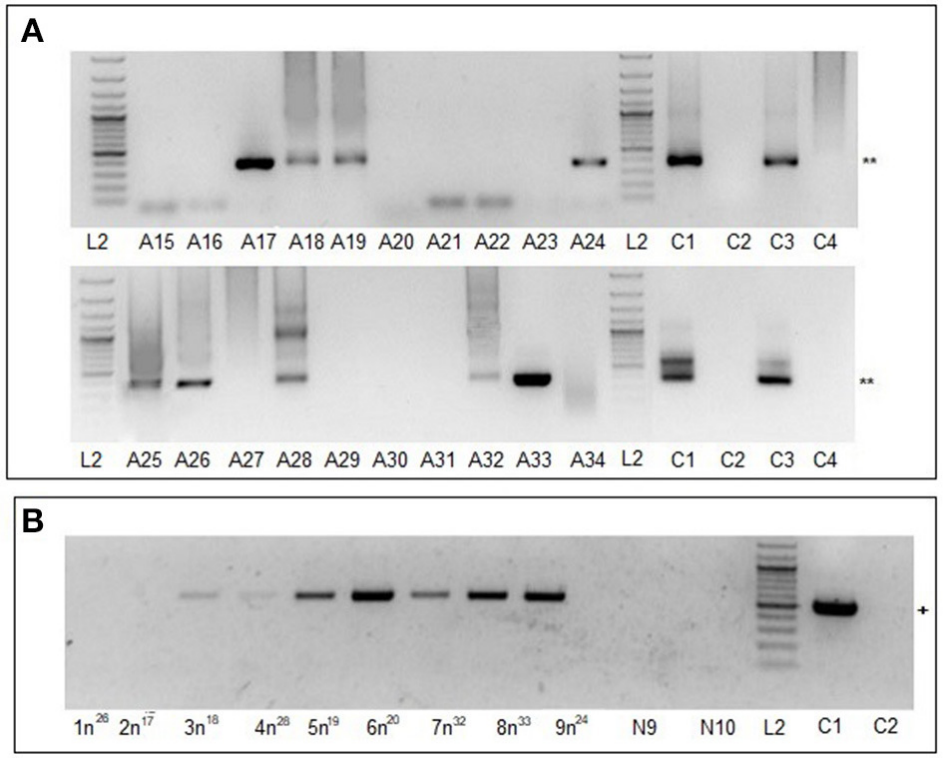
MHV68 detection in blood samples from mice infested with F0 females or F1 nymphs. **(A)** Lanes A15-A34, blood samples of 20 mice infested with infected F0 females; blood collected 15 days after tick infestation. ^**^Indicates MHV68 *ORF50* gene nested PCR product of 382 base pairs. **(B)** Lanes 1n^26^, 2n^17^, 3n^18^, 4n^28^, 5n^19^, 6n^20^, 7n^32^, 8n^33^, and 9n^24^, blood samples of mice infested with F1_i_ nymphs; N9, N10 blood samples of control mice infested with F1_c_ nymphs. ^+^Indicates MHV68 *M3* gene one step RT-PCR product of 520 base pairs. Lanes L2, C1–C4 as for Figure 1A.

To determine experimentally whether MHV68 can be transmitted vertically from infected adult ticks through the eggs to the following F1 generation, unfed adult female ticks (F0) were inoculated with 1.25 × 10^4^ PFU of MHV68 (0.5 μl) using a digital microinjector system. Following injection, the ticks were placed in a desiccator and maintained at room temperature and 90–95% relative humidity. Three weeks after virus injection, the female ticks (F0_i_) were allowed to infest 20 uninfected mice, 2 inoculated females together with 2 uninfected male ticks per mouse. As negative controls, non-inoculated females (F0_c_) were allowed to infest two uninfected mice. On day 15 post-infestation, when the last ticks to reach engorgement had dropped off, blood samples from the mice were collected and assayed for the presence of viral DNA by nested PCR. Nine of the 20 mice were positive (Figure 3A) (Table 2, Experiment 5). Following engorgement and drop off, the female ticks were maintained individually at room temperature under standard tick rearing conditions for 2 weeks until oviposition was complete. After ~50 days the eggs hatched into larvae (F1). In Experiment 6, F1_i_ larvae were fed on 9 uninfected mice (70–100 larvae per mouse), 46 days after hatching; each mouse received larvae from one PCR positive F0_i_ female. F1_c_ larvae from the uninfected control females (F0_c_) were fed on two uninfected mice. After completing feeding and drop off, the engorged F1 larvae recovered from each mouse were maintained as separate cohorts and allowed to molt to F1 nymphs (about 35 days).

**Table 2:** Vertical transmission of MHV68 in ticks.

**Experiment**	**Ticks**	**Molted ticks**	**Treatment**	**Number of mice tested**	**Mouse sample^a^**	**Number (%) virus positive mice**^b^			**Days after virus injection of F0 ticks**
						**Nested PCR**	**qPCR**	**RT-PCR**	
5	Adults (F0_i_)	n.a.	Injected with MHV68 and fed on uninfected mice	20	Blood	9 (45%)	n.t.	n.t.	38
5 (control)	Adults (F0_c_)	n.a.	Fed on uninfected mice	2	Blood	0 (0%)	n.t.	n.t.	n.a.
6	n.a.	Larvae (F1_i_)	Fed on uninfected mice	9	n.t.	n.a.	n.a.	n.a.	112
6 (control)	n.a.	Larvae (F1_c_)	Fed on uninfected mice	2	n.t.	n.a.	n.a.	n.a.	n.a.
7	n.a.	Nymphs (F1_i_)	Fed on uninfected mice	9	Blood	n.t.	9 (100%)	7 (77%)	252
					Lung	n.t.	9 (100%)	7 (77%)	
					Spleen	n.t.	9 (100%)	0 (0%)	
7 (control)	n.a.	Nymphs (F1_c_)	Fed on uninfected mice	2	Blood	n.t.	0 (0%)	0 (0%)	n.a.
					Lung	n.t.	0 (0%)	0 (0%)	
					Spleen	n.t.	0 (0%)	0 (0%)	
8	n.a.	Adults (F1_i_)	Fed on uninfected mice	9	Blood	0 (0%)	9 (100%)	n.d.	525
					Lung	8 (88%)	9 (100%)	7 (77%)	
					Spleen	6 (66%)	9 (100%)	4 (44%)	
8 (control)	n.a.	Adults (F1_c_)	Fed on uninfected mice	1	Blood	0(0%)	0 (0%)	n.d.	n.a.
					Lung	0 (0%)	0 (0%)	0 (0%)	
					Spleen	0 (0%)	0 (0%)	0 (0%)	

*n.a., not applicable; n.t., not tested*.

a *Samples collected from mice 15 days post-infestation*.

b *Nested PCR detects ORF50 gene; qPCR detects ORF65 gene representing genome equivalent number; RT-PCR detects M3 gene transcripts*.

**Figure 4:**
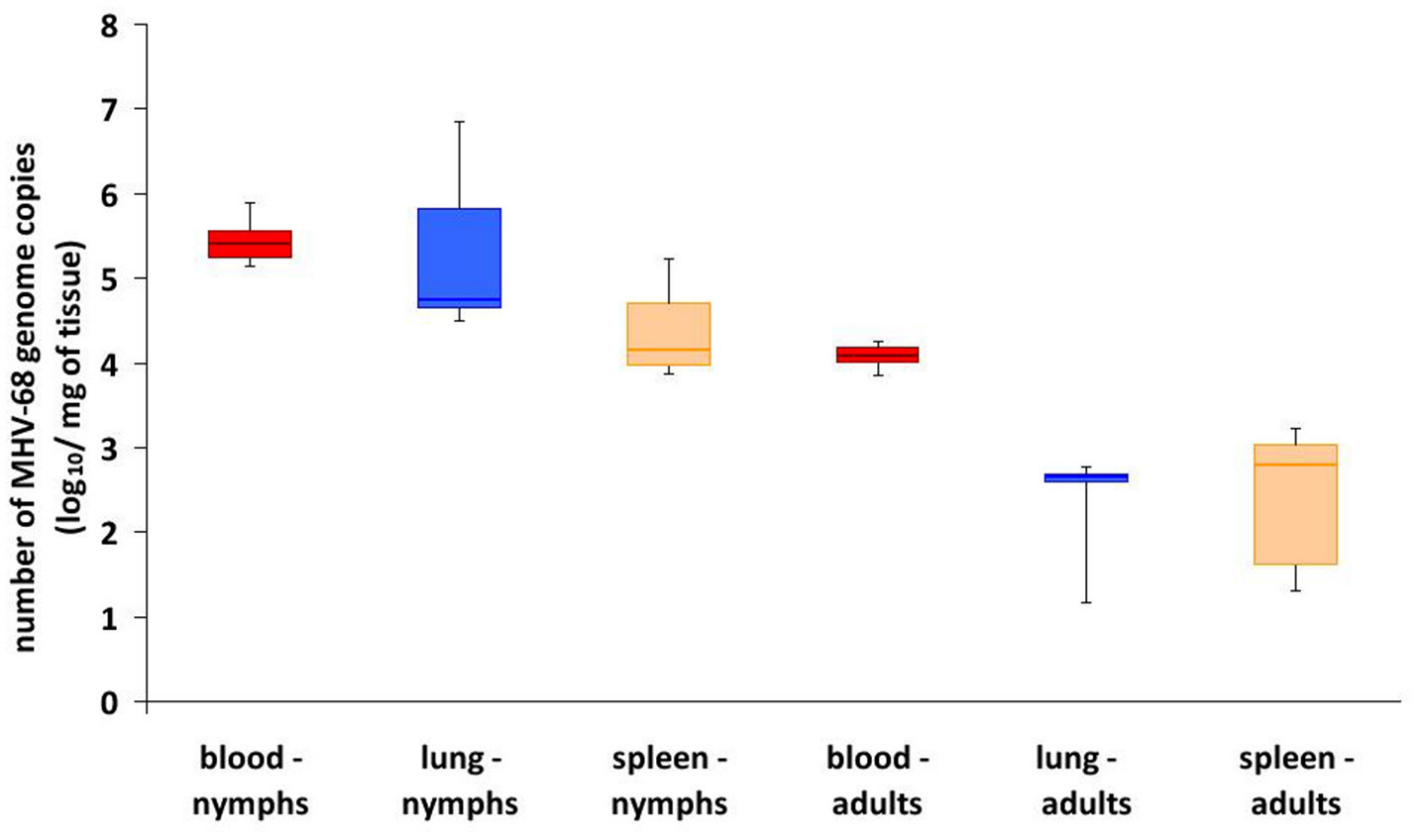
MHV68 genome load in organs of mice exposed to F1 infected ticks. Box and whisker plots of genome equivalent copies per ml blood or mg lung or spleen from mice 15 days after infestation with either F1_i_ nymphs or F1_i_ adult ticks. All controls were negative (see Table 2).

In Experiment 7, F1 nymphs were maintained for a further 81 days under standard conditions as separate cohorts, and then fed on uninfected mice (20 nymphs per mouse). On day 10, when the last F1 nymphs had completed engorgement and drop off, samples of blood, lung, and spleen from the mice exposed to F1 nymphs were collected and analyzed by quantitative real-time PCR (qPCR) based on number of virus genome equivalents (GE). Blood samples from all 9 mice exposed to F1_i_ nymphs were positive, with 1.4–7.8 × 10^5^ GE/ml, whereas the 2 mice exposed to F1_c_ nymphs were negative. Similarly, lung and spleen samples from the mice exposed to F1_i_ nymphs were all positive, ranging from 3.1 × 10^4^ to 7.2 × 10^6^ and 7.5 × 10^3^ to 1.7 × 10^5^ GE/mg, respectively, whereas the mice exposed to F1_c_ nymphs were negative (Table 2, Figure 4). Using nested RT-PCR, transcription of the M3 protein gene was detectable in blood samples (Figure 3B) and lungs (data not shown) of seven mice exposed to F1_i_ nymphs but not in the control mice. However, expression of the *M3* gene was not detected in spleen of any of the mice.

**Figure 5:**
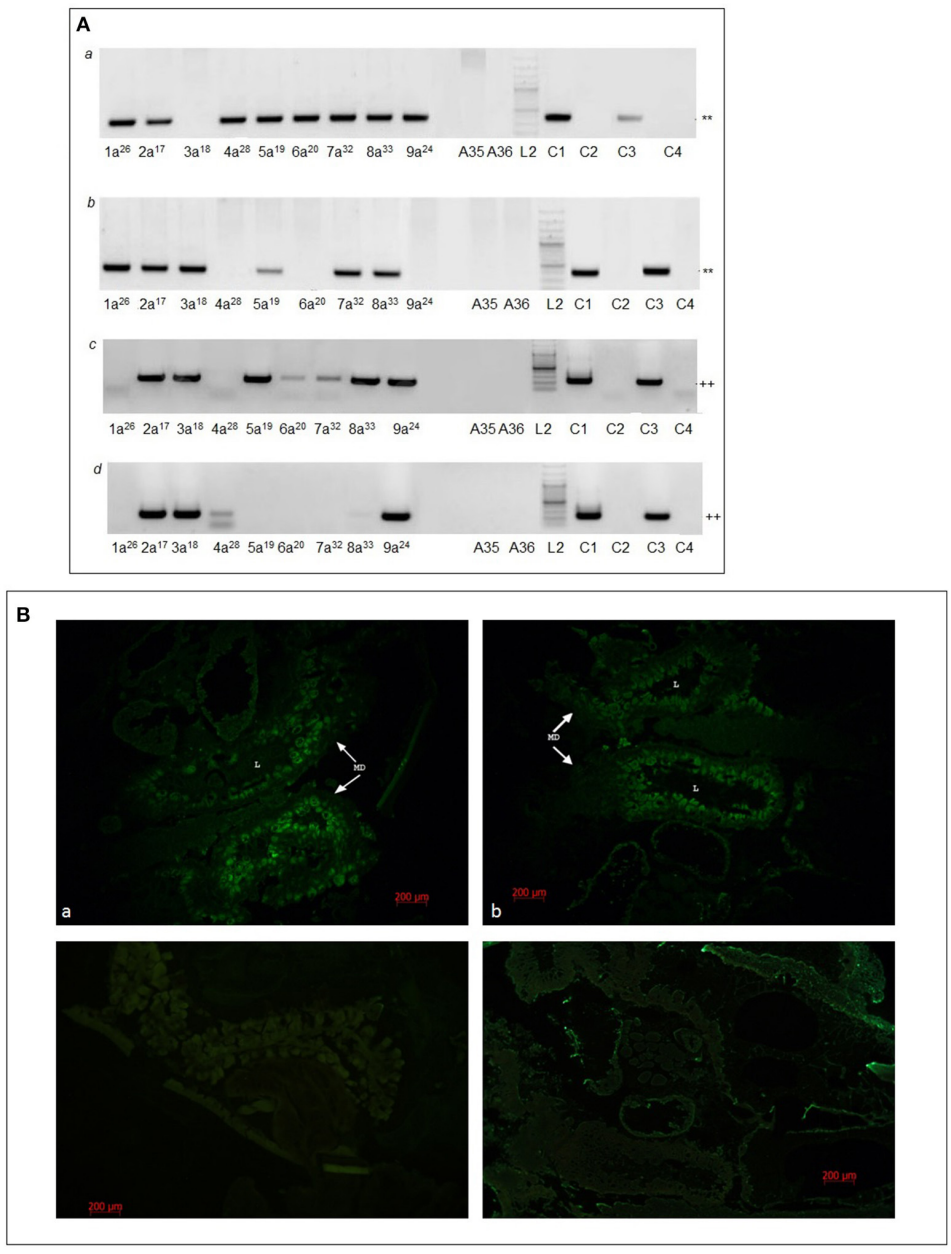
MHV68 detection in lung and spleen samples from mice infested with F1_i_ adults and in F1_i_ female ticks. **(A)** Lung **(a,c)** and spleen **(b,d)** samples of mice infested with F1_i_ adults examined by nested PCR **(a,b)** and RT-PCR **(c,d)**. Lanes 1a^26^, 2a^17^, 3a^18^, 4a^28^, 5a^19^, 6a^20^, 7a^32^, 8a^33^, and 9a^24^ samples of mice infested with F1_i_ adult ticks; A35, A36, samples of control mice infested with F1_c_ adults. Lanes L2, C1–C4 as for Figure 1A. ^**^ Indicates MHV68 *ORF50* gene nested PCR product of 382 base pairs; ^++^ indicates MHV68 *M3* gene nested RT-PCR product of 241 base pairs. **(B)** Semi-thin sections of frozen whole body of F1_i_ females fed for 4 days. **(a,b)** F1_i_ tick from mouse 3a^18^ and 5a^19^ stained with anti-MHV68 rabbit polyclonal serum; **(c)** uninfected tick (F6 generation of breeding) stained with anti-MHV68 rabbit polyclonal serum; **(d)** F1_i_ tick from mouse 3a^18^ stained with rabbit polyclonal serum against PB1-F2 protein of influenza virus A (H1N1) (negative control). MD, cells of midgut diverticula; L, lumen of midgut diverculum. Scale bar, 200 μm.

After engorgement and drop off, F1 nymphs were maintained under conditions that allowed molting to F1 adults (~6 months). Finally, in Experiment 8, after ~70 days of starvation, F1 adults were placed on uninfected mice (one female and one male per mouse). On day 15 days post-infestation, when the all ticks had dropped off, blood, lung, and spleen samples were collected from the mice. Screening by nested PCR was negative for all blood samples; however, qPCR identified MHV68 genome in blood samples of all mice exposed to F1_i_ adults with 7.0 × 10^3^ to 1.8 × 10^4^ GE/ml, whereas blood samples of mice exposed to F1_c_ adults remained negative (Table 2, Figure 4). Screening by nested PCR identified MHV68 in lung samples of eight (Figure 5A, *a*) and spleen samples of six mice (Figure 5A, *b*), whereas samples of both organs of the control mice remained negative (Table 2). qPCR quantification detected MHV68 in lung and spleen of all mice, ranging from 1.5 × 10^1^ to 6.0 × 10^2^ and 2.0 × 10^1^ to 1.7 × 10^3^ GE/mg, while lung and spleen of control mice were negative (Figure 5A). Further, transcription of the *M3* gene was detected by nested RT-PCR in lung samples of seven mice (Figure 5A, *c*) and in spleen samples of four mice (Figure 5A, *d*), whereas samples of the control mice remained negative. The duration between feeding artificially infected F0_i_ female ticks on uninfected mice and the detection of MHV68 DNA (in the blood, lungs, and spleen) and MHV68 *M3* gene transcripts (in lungs and spleen) in mice exposed to their F1_i_ adult progeny was ~525 days (Table 2). The number of GE in samples from mice exposed to F1_i_ nymphs was consistently higher than for F1_i_ adults (Figure 4), and was statistically significant for blood (χ^2^ = 7.37, d.f. = 1, *p* < 0.01).

Immunofluorescence staining of histological sections of whole ticks with rabbit anti-MHV68 polyclonal serum identified viral antigen in the intestines of two F1_i_ adult females that had fed 4 days on uninfected mouse 3a^4A^ or 5a^5A^ (Figure 5B). Specific staining was not detected in the salivary glands or other organs.

### Trans-stadial survival of virus: evidence of pathogenesis in ticks

In the experiments in which MHV68 DNA was detected in ticks fed on infected mice, there tended to be a lower survival rate of virus exposed ticks compared with uninfected ticks during molting (Table 3). Statistical logistic regression analysis of the data showed significantly fewer nymphs molted to adults (χ^2^ = 6.89, d.f. = 1, *p* = 0.01) that engorged on virus exposed mice (Scale promotor = 1.109) compared with control mice (Scale promotor = 1.0). The difference was not explained by feeding success as there was no significant difference in the numbers of uninfected nymphs that engorged on control uninfected mice compared with infected mice (χ^2^ = 0.02, d.f. = 1, *p* = 0.89). In Experiments 3 and 4 using nymphs, the feeding success was greater in Experiment 4, undertaken in the summer (mean engorged “infected” and “uninfected” nymphs, 71.7%) compared with Experiment 3 undertaken in the winter (mean engorged 48.1%). This difference in feeding success may have been due to physiological differences in the ticks. Interestingly, no difference was observed in the molting survival of ticks infected by vertical transmission.

**Table 3:** Differences in molting efficiency of larval (A) and nymphal (B) ticks feeding on control, uninfected, and MHV68 infected mice.

**Experiment^a^**	**Mice**	**No. of engorged larvae**	**No. of molted larvae to nymphs**	**%**
**A**				
2	MHV68 infected	613	172	28.0^ns^
	Control	56	20	35.7
**Experiment**^a^	**Mice**	**No. of engorged nymphs**	**No. of molted nymphs to adults**	**%**
**B**				
3	MHV68 infected	50	24	48.0
	control	27	17	63.0
4	MHV68 infected	99	72	72.7
	Control	30	28	93.3
Total	MHV68 infected	149^ns^	96	64.4^*^
	Control	57	45	78.9

a *See Table 1*.

ns, not significantly different from control;

* *significantly different, p = 0.01*.

## Discussion

Herpesviruses typically have a narrow host range and are transmitted by contact with infected body fluids such as saliva, urine, tears, and breast milk. Hitherto, vector-borne transmission of herpesviruses has not been demonstrated although infectious bovine rhinotracheitis virus (*Bovine herpesvirus 1*) was isolated from three separate collections of the argasid tick, *Ornithodoros coriaceus*, over a 3-year period (Taylor et al., 1982). The ticks were collected from deer bedding areas in the Sierra Nevada Mountains, USA, where antibodies to the virus were detected in both deer and cattle; virus transmission studies were not reported. Similarly, the report of MHV68 PCR positive ticks collected from wild caught lizards in Slovakia did not demonstrate virus transmission (Ficová et al., 2011). However, MHV68 detection in field-collected *D. reticulatus* ticks included isolation of infectious virus from the salivary glands, ovaries and midgut of these ticks (Kúdelová et al., 2015). More recently, transcripts of the early-late *M3* gene of MHV68 were detected in 10 of 11 questing field-collected *D. reticulatus* ticks, with 2.2 × 10^4^-8.6 × 10^6^ GE/tick (Kúdelová et al., 2017). Although MHV68 antibodies have been recorded in free-living animals and humans, a common explanation is one of antigenic cross-reactivity with other gammaherpesviruses (Hricová and Mistríková, 2007). Natural host entry by MHV68 is believed to occur via the upper respiratory tract (Milho et al., 2009). Nevertheless, no evidence of direct horizontal transmission was found between female laboratory mice or between female bank voles housed together in captivity, and the significance of milk-borne transmission and female to male sexual transmission, demonstrated experimentally in laboratory mice, have yet to be determined (Raslova et al., 2001; François et al., 2010, 2013).

We undertook a rational sequence of experiments to determine if ticks can transmit MHV68 and whether the virus shows the characteristics of an arbovirus (Nuttall et al., 1994; Kuno and Chang, 2005). First, we artificially infected ticks by injecting adult females (the largest tick stage) with mammalian cell culture-grown virus. Infectious virus survived in the ticks for at least 120 days, and viral DNA was detected in the tick salivary glands and in blood of mice on which the virus-injected ticks fed (Experiment 1). Based on these positive results, we tested whether ticks can be infected naturally, by feeding on infected mice (Experiments 2–4). The results showed that MHV68 DNA, acquired by larvae and nymphs feeding on infected mice, was subsequently transmitted by nymphs and adults (respectively, after molting) to uninfected mice. In contrast to blood-feeding insects, ticks feed exclusively on blood and ixodid species take only one bloodmeal (increasing their body weight ~100-fold) at each developmental stage (3 active instars: larva, nymph, adult). Three-host ixodid ticks, such as *I. ricinus*, drop off the host after completing engorgement and hide in the undergrowth where they molt or lay eggs. Molting involves extensive tissue breakdown and remodeling, which ticks survive depending on how successfully they have fed and the environmental conditions; arboviruses survive the hostile environment within the metamorphosing tick by infecting cells not subjected to extensive tissue histolysis (Nuttall et al., 1994; Kuno and Chang, 2005). The results of feeding ticks on infected mice indicate that MHV68 survived molting and was transmitted by the subsequent developmental stage.

Some arboviruses, such as TBEV and African swine fever virus (ASFV), are maintained vertically in ticks although the levels of transovarial and trans-sexual transmission typically are <1.0% in *I. ricinus* (Rennie et al., 2001; Danielová et al., 2002). Evidence of vertical transmission was found for MHV68: both F1_i_ nymphs and F1_i_ adults transmitted MHV68 during feeding on uninfected mice (Experiments 7 and 8). Remarkably, F1_i_ adult ticks transmitted MHV68 >500 days after the initial infection of the F0 adult females, and all mice on which the ticks fed showed evidence of infection. The apparent efficiency of vertical transmission was likely augmented by horizontal transmission in which uninfected ticks acquired MHV68 while co-feeding with infected ticks, either from viremic mice or via non-viremic transmission (Tibbetts et al., 2003; Kuno and Chang, 2005). Immunomodulation at the site of tick feeding, including inhibition of type 1 interferon and reprogramming of dendritic cells by tick saliva, may promote acquisition and co-feeding transmission of MHV68 by ticks (Kazimírová et al., 2017; Lawler and Stevenson, 2017). Detection of viral antigen in the midgut of F1_i_ adult females at 3 days of feeding is consistent with augmentation of infection by an infected bloodmeal. Heparan sulfate glycosaminoglycan expression has been recorded in the gut and ovaries of ixodid ticks (Onofre et al., 2003). Given that heparan binding is a key event during infection by cell free MHV68, and that bloodmeal digestion in ticks is intracellular and does not involve extracellular acidic proteolysis of gut contents, conditions in ticks appear to meet at least some of the requirements for MHV68 infection, for example, if a tick ingested infected B cells (Gillet et al., 2009; Sonenshine and Roe, 2013).

Acute MHV68 infection in lungs and spleen of outbred mice is cleared within 9–15 days; although the dynamics of acute phase replication are delayed at low doses (e.g., 0.1 PFU), peak titer and subsequent frequency of latently infected cells are unaffected by dose (Tibbetts et al., 2003). Hence we examined blood, lung, and spleen at 15 days after infestation with F1 ticks, using different PCR methods to detect the presence of MHV68 DNA (nested PCR), to quantify the virus load (qPCR), and/or to determine evidence of viral replication (RT-PCR). Differences in results obtained with the different PCR methods suggest differences in their sensitivity. Initially, nested PCR targeting *ORF50* was selected as this assay was developed to detect one GE of MHV68 per 250 ng tick DNA (Kúdelová et al., 2015). However, subsequent comparisons with mouse samples revealed the detection limit of the nested *ORF50* PCR assay was at least 1,000-fold less than that of qPCR for *ORF65* and RT-PCR for *M3*, indicating that some studies (Experiments 2–4) probably underestimated the frequency of virus transmission. By qPCR, MHV68 *ORF65* was detected in all samples from all mice exposed to F1_i_ nymphs and adults (Experiments 7 and 8), although significantly more MHV68 GE was detected in mice exposed to nymphs compared with adults (10^5^ vs. 10^4^ in blood, 10^4^ vs. 10^2^ in lung, and 10^4^ vs. 10^2^ in spleen, respectively). This indicates that the 20 F1_i_ nymphs infesting each mouse transmitted more MHV68 than the 2 adult females and 2 males/mouse, which is unsurprising especially given that adult male *I. ricinus* feed little if at all. In mice exposed to F1_i_ nymphs (Experiment 7), evidence of replicating virus was detected in blood and lung but not in spleen, and in mice exposed to F1_i_ adults (Experiment 8), fewer spleen samples showed evidence of virus replication compared with lung samples. Virus replication (detected as *M3* gene transcripts) indicates that an acute infection of mice was ongoing at the time of sampling, which was most apparent in blood and lung (Ebrahimi et al., 2003). In spleen, MHV68 replication is usually limited and occurs a short time before virus latency is established in B cells, predominantly in the spleen (Speck and Ganem, 2010).

For insect-borne arboviruses, there is some evidence that arboviral infection is detrimental to the arthropod (insect) vector whereas tick-borne viruses appear to have little effect on their tick vectors with the notable exception of ASFV (Nuttall et al., 1994; Kuno and Chang, 2005). ASFV has been reported to cause mortality of up to 73% when infecting its argasid tick vector, *Ornithodoros* species, although a number of reports have not found significant virus-induced mortality (Rennie et al., 2000; Kleiboeker and Scoles, 2001). Hence the apparent pathogenic effect of MHV68 in *I. ricinus* is unusual for a tick-borne arbovirus but not unprecedented. However, there was no evidence vertical transmission of MHV68 had a detrimental effect on ticks. The apparent efficiency of vertical transmission may at least partly explain the unusually high incidence of MHV68 detected in ticks collected in the field. Of 432 adult *D. reticulatus* collected at two sites in southwestern Slovakia from 2011 to 2014, 45% female ticks and 26% male ticks were PCR positive for MHV68 *ORF50*, and infectious virus was isolated from salivary glands, midgut, and ovaries of 2/5 female ticks (Kúdelová et al., 2015). More recently, evidence was reported of virus in free-living immature and adult *I. ricinus* ticks (unpublished data) and 38% (18/47) questing adult *Heamaphysalis concinna* ticks, with 2 × 10^2^-9.6 × 10^3^ GE detected in the latter species (Vrbová et al., 2016).

Detection of MHV68 in *I. ricinus* ticks collected from free-living green lizards (*L. viridis*) in the Slovak Karst National Park in 2007 raises questions about the role of lizards in the natural history of MHV68 (Ficová et al., 2011). Our re-examination of the published data suggests that lizards are not susceptible to MHV68. Of the total 799 ticks collected from 89 green lizards, there were 1.5% positive nymphs and 3.3% positive larvae. If lizards are competent hosts of MHV68, more nymphs than larvae should be infected, as found with other tick-borne pathogens (Václav et al., 2011). Based on our experimental observations, the most likely source of MHV68 in *I. ricinus* ticks removed from lizards was through vertical transmission from ticks previously fed on infected rodents.

MHV68 and related viruses have been reported in Slovakia, Czech Republic, France, Northern Ireland, Germany, and England. Most isolates have been from the yellow-necked field mouse, *A. flavicollis*, although the first reported isolations were from bank voles, *M. glareolus*. In addition to these two rodent species, MHV-68 strains have been detected by PCR in a further 3 rodent species, and MHV68 neutralizing antibodies have been reported in 13 mammalian species (Mistríková et al., 2000a,b; Nash et al., 2001). This apparent ability to infect a diversity of mammalian species is typical of an arbovirus but unusual for a herpesvirus. Generally, the natural host range of individual herpesviruses is highly restricted; indeed, most herpesviruses were thought to have evolved in association with a single host species although phylogenetic analysis of the subfamily *Gammaherpesvirinae* suggests evolution both by cospeciation with host lineages and by transfer between widely distinct hosts (Ehlers et al., 2008). While bats and humans are considered potential superspreaders to other mammalian taxa (Escalera-Zamudio et al., 2016), our data suggest ticks are potential megaspreaders. Tick-borne transmission is a plausible explanation for the detection of MHV68 neutralizing antibodies in fallow deer, red deer, wild boar, and sheep, all of which are commonly infested by *I. ricinus*. Indeed, the pattern of seroprevalence for MHV68 is similar to the patterns for TBEV and *B. burgdorferi*, which reflect the hosts on which their tick vector (*I. ricinus*) feeds, and the susceptibility to infection of the various species (Kozuch et al., 1990; Trávnicek et al., 2003).

Evidence MHV68 may infect humans was first reported in 20 personnel working with MHV68 or who were exposed to small rodents at the Institute of Virology, Bratislava and the Department of Microbiology and Virology of Comenius University, Bratislava (Mistríková et al., 2000a). Further studies reported antibodies to MHV68 in 4 cohorts, including hunters and a group of patients associated with infectious diseases (Hricová and Mistríková, 2007). The possibility that these reports were the result of antigenic cross reactions with human herpesviruses was examined in a study of 330 sera from patients of different hospitals in Slovakia with anonymous diagnoses, and from employees of the Department of Microbiology and Virology of Comenius University, Bratislava (Hricová and Mistríková, 2007). The results and antibody titers were the same using virus neutralization tests (VNT) and immunofluoresence assays (IFA), both indicating an antibody prevalence of 4.5%; ELISA indicated higher antibody titers and a higher prevalence, 16.1%. Cross reactivity was detected by ELISA with Epstein Barr virus though not with herpes simplex virus-1 and human cytomegalovirus antigens. Human sera positive for Epstein Barr virus (3 sera), herpes simplex virus-1 (2 sera) and human cytomegalovirus (5 sera) did not neutralize MHV68 in the VNT (Mistríková et al., 2000a). Hence antigenic cross-reactivity between MHV68 and human herpesviruses was detected by ELISA but not using VNT. Further work is needed to determine whether MHV68 can infect humans and, if so, whether the virus is transmitted to humans by *I. ricinus* ticks.

## Author contributions

VHa, MK, and MS conceived and designed the experiments. MK, ZH, PP, IŠ, PBa, PBe, MS, MV, VHo and MK, performed the experiments. MK, VHa, PN, and RH analyzed the data. PN, MK, and VHa wrote the paper.

### Conflict of interest statement

The authors declare that the research was conducted in the absence of any commercial or financial relationships that could be construed as a potential conflict of interest.
